# Subjective Outcome Evaluation of the Project P.A.T.H.S.: Qualitative Findings Based on the Experiences of Program Participants

**DOI:** 10.1100/tsw.2007.126

**Published:** 2007-05-29

**Authors:** Daniel T. L. Shek, Rachel C. F. Sun

**Affiliations:** ^1^ Centre for Quality of Life, Hong Kong Institute of Asia-Pacific Studies, The Chinese University of Hong Kong, Hong Kong; ^2^ Kiang Wu Nursing College of Macau, Macau; ^3^ Social Welfare Practice and Research Centre, Department of Social Work, The Chinese University of Hong Kong, Shatin, Hong Kong

**Keywords:** subjective outcome evaluation, open-ended questions, qualitative data, positive youth development, Hong Kong

## Abstract

A total of 52 schools participated in the Experimental Implementation Phase of the Project P.A.T.H.S. After completion of the Tier 1 Program, 8,057 students responded to a Subjective Outcome Evaluation Form (Form A) to assess their views of the program, instructors, and perceived effectiveness of the program. Based on the schools' evaluation reports, results of secondary data analyses on four open-ended questions showed that: (a) students felt that they had learned things at the personal, interpersonal, familial, and societal levels; (b) they appreciated the program design, instructors' performance, learning process, and program effectiveness; (c) they generally had positive comments on instructors attitude and teaching process; and (d) they made some suggestions on how the program and its implementation could be improved. The present study, based on qualitative data of subjective outcome evaluation, provides additional support for the effectiveness of the Tier 1 Program of the Project P.A.T.H.S. in Hong Kong.

## INTRODUCTION

In human services, the importance of involving the service users or program participants in evaluation is advocated, and thus subjective outcome evaluation becomes popularly used to capture the viewpoints of the participants. In this regard, client satisfaction surveys are commonly used as feedback for transforming services to meet the users' needs for planning and administration purposes, or simply used as an indicator of program effectiveness from the participants' perspective for research purposes. The commonly used approach is to develop closed-ended rating scale items to quantify client satisfaction. For example, standardized rating scales, such as the Medical Interview Satisfaction Scale[1], Consumer

Satisfaction Questionnaire[2], and Client Satisfaction Questionnaire[3] were developed to gauge client satisfaction and perceived helpfulness of the program.

Although objective client satisfaction questionnaires with adequate psychometric properties have been devised, there are several criticisms regarding the use of quantitative strategy to assess client satisfaction. The first criticism is the limitation based on the restricted response format associated with closed-ended rating scales. In the context of subjective outcome evaluation in the health context, Avis et al.[4] remarked that "although patient satisfaction questionnaires provide apparent ease of measurement, asking patients to respond to questionnaire items only serves to channel their concerns into avenues pre-defined by the providers, rather than allow free expression of their perceptions and experiences" (p. 320). In addition, Williams et al.[5] argued that the closed-ended questions would limit the clients' expression of negative experiences, and thus "high satisfaction ratings do not necessarily mean that patients have had good experiences in relation to the services; rather, expressions of satisfaction may more often reflect attitudes such as 'they are doing the best they can', or 'it's not really their job to do…'" (p.1358). Similarly, Williams[6] queried whether the subjective outcomes obtained by the quantitative method could reveal the underlying perceptions and experiences of the respondents, and noted "consequently, inferences made from their results may misrepresent the true beliefs of service users" (p.515).

Based on the above criticisms, it is argued that it is important to adopt a qualitative approach to explore the clients' positive and negative experiences of services and their perceptions of the improvement of services that could be made. Other researchers also supported the use of qualitative research methods to elicit the clients' subjective experiences and perceptions of services[7], in order to complement the quantitative findings on client satisfaction[8] and to yield valuable information about the functioning of the services[9]. In his discussion of how the objectivity of client satisfaction could be improved, Royse[10] argued that "use at least one open-ended question to give consumers the opportunity to inform you about problems you did not suspect and could not anticipate" (p. 265). Weinbach[11] also argued that "it is also a good idea to have a mixture of fixed-alternative and open-ended items in a client satisfaction survey" (p. 126).

One common qualitative approach to a client satisfaction survey is to use open-ended questions to assess the subjective perceptions of the program participants[12]. These open-ended questions can be used to further explore the clients' positive experiences, such as the aspects of services they felt satisfied with or appreciated. Further, along the satisfaction-dissatisfaction continuum, the open-ended questions can be designed to allow clients to express their dissatisfaction of the services, any negative experiences, and to point out which aspects of the services could be improved. Moreover, various types of open-ended questions can also be asked in response to different research questions, such as to examine the helpfulness of the program by asking the respondents the gains perceived after attending the program, or to investigate the qualities of the program through asking the respondents their perceptions on the skills of the workers. Research studies have shown that the data collected by the open-ended questions could generate more detailed and precise information on the clients' opinions on the services in terms of good and bad facets, as compared with the data collected by the closed-ended questions[13]. Therefore, using open-ended questions is pertinent in subjective outcome evaluation.

In the review of literature, there were many studies asking respondents to answer open-ended questions in self-administration questionnaires. Some of them used one open-ended question as part of a questionnaire survey on client satisfaction. For example, "What could Vocational Rehabilitation Services do to improve your satisfaction with the program?" was asked in a study in the U.S.[14], and "If you are not satisfied (with medical care), what are the reasons of this?" was asked in a study in Lithuania[15], in order to draw conclusions on the concrete aspects of a program that could be improved. Other studies used two to three open-ended questions to explore clients' subjective views, including perceived factors contributing to the clients' positive and negative feelings about the termination of the psychotherapy in Israel[16]; perceived factors contributing to the Finnish womens' satisfaction and dissatisfaction on breast cancer screening[17]; and the perceived best and worst aspects of eating-disorder consultation services in New Zealand and the areas that could be improved[18]. In these studies, as the open-ended questions were designed to elicit respondents' answers in areas of satisfaction, dissatisfaction, and improvement of services, these qualitative data did not just add support to the high satisfaction rates obtained from the items with fixed-response choices (quantitative data), but they also illuminated other aspects (e.g., dissatisfaction) that had not been tapped by the quantitative methods. All in all, the above studies highlighted the importance of using the qualitative approach and were in favor of using open-ended questions to explore clients' subjective views because it is a practical means to collect particular information of client satisfaction and dissatisfaction, and give insights to the future program improvements.

The Project P.A.T.H.S. is a positive youth development program that attempts to promote holistic development in adolescents in Hong Kong. The students participating in the Tier 1 Program (Secondary 1 Level) were required to respond to the Subjective Outcome Evaluation Form (Form A), where both quantitative and qualitative data were collected. Based on the evaluation reports on the consolidated subjective outcome evaluation profile of 52 participating schools, Shek and Ma[19] aggregated the quantitative subjective outcome evaluation data and reconstructed an overall profile of the perceptions and experiences of the program participants. The quantitative evaluation findings basically revealed that the program participants had positive perceptions of the program, instructor, and the effectiveness of the program. Although the findings reported by Shek and Ma[19] are very encouraging, it would be helpful if qualitative findings based on the open-ended questions could be examined. In the present paper, qualitative analyses of the school evaluation reports submitted based on a general qualitative orientation[[20] were conducted to answer the following research questions:

1. What were the most important things that student learned in the program?
2. What were the things the students appreciated most in the program?
3. What comment did the students have on the instructor?
4. Which aspects of the program required improvement?

## METHODS

### Participants and Procedures

There were 52 schools joining the Experimental Implementation Phase of the Project P.A.T.H.S. (Positive Adolescent Training through Holistic Social Programmes). In the Tier 1 Program, 29 schools adopted the full program (i.e., 20-h program involving 40 units) and 23 schools adopted the core program (i.e., 10-h program involving 20 units). After the program was completed, the participants were invited to respond to a subjective outcome evaluation questionnaire. A total of 8,057 students (mean = 154.94 students per school, ranged from 37 to 212) responded to the Subjective Outcome Evaluation Form (From A) developed by the Research Team. As the Project P.A.T.H.S. was financially supported by the Hong Kong Jockey Club Charities Trust, each participating school was required to submit an evaluation report, together with the consolidated subjective outcome evaluation profile of the school, to the funding body. In other words, the workers were expected to conduct program evaluation as part of their professional practice. To facilitate the program evaluation, the Research Team developed an evaluation manual with standardized instructions for collecting the subjective outcome evaluation data[21]. In addition, adequate training was provided to the workers during the 20-h training workshops on how to collect and analyze the data collected by Form A.

The data collection was normally carried out at the last session of the program, and administered by school teachers or social workers. On the day when the evaluation data were collected, the purpose of the evaluation was mentioned and the confidentiality of the data collected was repeatedly emphasized to all of the students. The students were asked to indicate their wish if they did not want to respond to the evaluation questionnaire (i.e., "passive" informed consent were obtained from the students). All participants responded to all scales in the evaluation form in a self-administration format. Adequate time was provided for the participants to complete the evaluation form. In terms of the number of participants in the qualitative analyses, it can be regarded as sufficient.

## Instruments

The Subjective Outcome Evaluation Form (Form A) was designed by Daniel Shek and Andrew Siu[21]. Broadly speaking, there are several parts in this evaluation form as follows:

- Participants' perceptions of the program, such as program objectives, design, classroom atmosphere, interaction among the students, and the respondents' participation during class (10 items).
- Participants' perceptions of the workers, such as the preparation of the instructors, professional attitude, involvement, and interaction with the students (10 items).
- Participants' perceptions of the effectiveness of the program, such as promotion of different psychosocial competencies, resilience, and overall personal development (16 items).
- The extent to which the participants would recommend the program to other people with similar needs (1 item).
- The extent to which the participants would join similar programs in the future (1 item).
- Overall satisfaction with the program (1 item).
- Things that the participants learned from the program (open-ended question).
- Things that the participants appreciated most (open-ended question).
- Opinion about the instructor(s) (open-ended question).
- Areas that require improvement (open-ended question).

## Data Analyses

After receiving the schools' evaluation reports from the funding body, the Research Team aggregated the data to "reconstruct" the overall profile based on the subjective outcome evaluation data. The present study focused on the four open-ended questions and conducted secondary data analyses of the responses to these questions. The data were analyzed using general qualitative analyses techniques[22] by a registered social worker and a colleague with a master degree in Social Work. There were three steps in the process. First, relevant raw codes were developed for words, phrases, and/or sentences that formed meaningful units (i.e., raw responses level). Second, the codes were further combined to reflect higher-order attributes (i.e., category of codes level). Third, the categories of codes were further analyzed to reveal the broader categories at the thematic level. For example, the response that students had learned "optimism" at the raw response level could be subsumed under the category of "ways to face adversity", which can be further subsumed under the broad theme of "personal competence" (see Table 1). To ensure that the findings are auditable, the raw data and categorized data were kept in a systematic filing system.

In qualitative study, two important issues are whether the researchers are conscious of their own biases and how biases can be minimized. In the present context, because the researchers designed the program in the Project P.A.T.H.S., they would expect the program to be effective. As such, how to minimize the possible biases involved is an important issue to be considered. In order to ensure the reliability of the coding, both intra- and inter-rater reliability on the coding was calculated. For intrarater reliability, two colleagues who coded the responses were invited to individually code 20 randomly selected responses for each question. For inter-rater reliability, two research assistants (one with a bachelor's degree and one with a master's degree) coded 20 randomly selected responses for each question (except Question 2 where there were 21 randomly selected responses) without knowing the original codes given at the end of the scoring process with reference to the codes finalized by the researchers.

**TABLE 1**
**Summary of the Students' Responses to the Question "What were the Most Important Things that Students Learned in the Program?"**

| Level of Competence | Subcategory | Item | N | N in Subcategory |
|---|---|---|---|---|
| Societal level | Social norms | Following customs | 3 | |
| | | Following social norms | 4 | |
| | | Following rules | 3 | 10 |
| | Society and social responsibility | Knowledge about China | 11 | |
| | | Citizen's responsibilities | 3 | |
| | | Contribution to society | 5 | 19 |
| Familial level | | Communication with family members | 12 | 12 |
| Interpersonal level | General interpersonal competence | Team spirit | 23 | |
| | | Interpersonal relationship | 45 | |
| | | Making friends | 31 | |
| | | Getting along with healthy peers | 25 | 124 |
| | Specific interpersonal competence | Mutual help | 3 | |
| | | Punctuality | 1 | |
| | | Caring for others | 19 | |
| | | Communication | 21 | |
| | | Apology | 1 | |
| | | Politeness | 5 | |
| | | Acceptance | 3 | |
| | | Respect | 19 | 72 |
| Personal level | Cherishing life | To cherish life | 21 | |
| | | To treasure people and things | 10 | 31 |
| | Reflection | Reflection | 12 | |
| | | Wisdom of existence | 1 | 13 |
| | Cognitive competence | Rational thinking and analytical thinking | 13 | |
| | | Creativity | 1 | |
| | | Objectivity | 1 | |
| | | Problem solving | 2 | 17 |
| | Ways to face adversity | Resilience | 11 | |
| | | To think about future | 1 | |
| | | Be active | 5 | |
| | | Optimism | 3 | |
| | | Courage | 4 | |
| | | Toughness | 2 | 26 |
| | Positive self-image | Self-understanding | 33 | |
| | | Self-respect | 2 | |
| | | Self-confidence | 28 | |
| | | Personal growth | 7 | 70 |
| | Emotional competence | Emotional management | 43 | 43 |

**TABLE 1 (continued)**

| Level of Competence | Subcategory | Item | N | N in Subcategory |
|---|---|---|---|---|
| | Assertiveness and goal setting | Willing to express oneself | 4 | |
| | | Refusing negative influences | 4 | |
| | | Setting a specific goal | 12 | |
| | | Making choices wisely | 6 | |
| | | Self-determination | 2 | 28 |
| | Moral competence and virtues | Civic mindedness | 9 | |
| | | Personal ethics | 1 | |
| | | Fairness | 5 | |
| | | Equality | 1 | |
| | | To distinguish between right and wrong | 15 | |
| | | Sense of responsibility | 8 | |
| | | Integrity | 3 | |
| | | Seriousness | 1 | |
| | | Concentration | 5 | |
| | | Patience | 1 | 49 |
| Others | | Other opinions | 5 | |
| | | Opinions that were not directly related to what students had learned | 12 | 17 |
| | | **Total (N)** | **531** | |

*Note*: Twenty coded raw descriptors were randomly selected for examining intra- and inter-rater reliability. The raters were asked to code the randomly selected descriptors into four categories (i.e., societal level, familial level, interpersonal level, personal level) without knowing the original codes given.

## RESULTS

As shown in Table 1, a total of 531 meaningful units in five categories (i.e., societal, familial, interpersonal, personal levels of competence, and others) were formed to indicate the most important things that students learned in the program. Most of the respondents reported that they learned interpersonal competence, which could be broken into “general interpersonal competence” (N = 124, e.g., how to make friends) and “specific interpersonal competence” (N = 72, e.g., communication). In addition, learning regarding “positive self-image” (N = 70, e.g., self-understanding), “moral competence and virtues” (N = 49, e.g., to distinguish between right and wrong), and “emotional competence” (N = 43, e.g., emotional management) at the personal level was found. The percentages of intrarater agreement were 95 and 100%, and those of inter-rater agreement percentage were 100 and 100%.

The things that students appreciated most in the program are shown in Table 2. A total of 342 meaningful units in five categories (i.e., program design, instructors’ performance, learning process, program effectiveness, and others) were extracted. The three most appreciated areas were the “program content” (N = 76, e.g., rich or appropriate curriculum), “learning process” (N = 58, e.g., students were involved), and “program effectiveness” (N = 56, e.g., broadened learning areas). The percentages of intrarater agreement were 100 and 100%, and those of inter-rater agreement percentage were 85 and 80%.

**TABLE 2**
**Summary of the Students' Responses to the Question "What were the Things the Students Appreciated most in the Program?"**

| Area | Subcategory | Item | N | N in Subcategory |
|---|---|---|---|---|
| Program design | Content | Having clear teaching objectives | 5 | |
| | | Diverse program content | 16 | |
| | | Rich/appropriate curriculum | 36 | |
| | | Interesting curriculum and activities | 19 | 76 |
| | Format | Interesting teaching format | 6 | |
| | | Having discussion | 12 | |
| | | Having games | 12 | |
| | | Good teaching materials | 5 | 35 |
| Instructors' performance | Overall impression | Performance of instructors was good | 22 | 22 |
| | Teaching process | Instructors had good teaching and facilitating skills | 14 | |
| | | Instructors were involved | 16 | |
| | | Good relationship between instructors and students | 2 | |
| | | Instructors were willing to share | 4 | 36 |
| | Attitude | Good teaching attitude | 27 | |
| | | Instructors were conscientious | 13 | |
| | | Instructors were caring and concerned about students | 6 | 46 |
| Learning process | Learning process | Happy learning process | 3 | |
| | | Good atmosphere during the lessons | 17 | |
| | | Interactive | 6 | |
| | | Students were involved | 23 | |
| | | Satisfactory discussion process | 9 | 58 |
| | Learning arrangement | Appropriate learning arrangement | 4 | 4 |
| Program effectiveness | | Broadened learning areas | 25 | |
| | | Enhanced interaction and communication skills | 11 | |
| | | Enriched friendship and enhanced cooperation | 17 | |
| | | Built up courage | 3 | 56 |
| Others | | Other aspects appreciated | 9 | 9 |
| | | **Total (N)** | **342** | |

*Note*: Twenty-one coded raw descriptors were randomly selected for examining intra- and inter-rater reliability. The raters were required to code the randomly selected descriptors into four categories (i.e., program design, instructors' performance, learning process, program effectiveness) without knowing the original codes given.

As shown in Table 3, there were a total of 361 positive and negative comments made on the instructors by the students, which were classified into five categories (i.e., overall comment, attitude, teaching process, suggestion, and others). Most of the respondents commented on the instructors' attitude in the aspects of "relationship with students" (N = 64, e.g., kind and friendly) and "sense of responsibility" (N = 60, e.g., good preparation), as well as "teaching methods or skills" (N = 58) in the

**TABLE 3**
**Summary of the Students' Responses to the Question "What Comments did the Students have on the Instructor?"**

| Area | Subcategory | Item | N | N in Subcategory |
|---|---|---|---|---|
| Overall comment | | Overall comment was positive | 52 | |
| | | Having some good and bad points | 1 | |
| | | Performance of instructors was not good* | 2 | 55 |
| Attitude | Relationship with students | Kind and friendly | 34 | |
| | | Unfriendly* | 1 | |
| | | Good relationship with students | 2 | |
| | | Concerned about students | 14 | |
| | | Willing to offer assistance to students | 8 | |
| | | Understand students' feelings and needs/considerate | 5 | 64 |
| | Sense of responsibility | Active | 7 | |
| | | Responsible | 3 | |
| | | Serious | 16 | |
| | | Circumspect/attentive/careful | 14 | |
| | | Good preparation | 20 | 60 |
| | Attitude | Patient | 14 | |
| | | Involved | 24 | |
| | | Professional | 9 | 47 |
| Teaching process | Teaching methods/skills | Good teaching methods | 15 | |
| | | Diverse teaching methods | 3 | |
| | | Rigid/inflexible teaching methods* | 3 | |
| | | Boring/dull/atmosphere was not good* | 9 | |
| | | Clear and detailed explanation | 9 | |
| | | Unclear explanation* | 2 | |
| | | Facilitate students' learning | 17 | 58 |
| | Interaction with students | Lack of responses/interaction* | 4 | |
| | | Much interaction with students | 4 | 8 |
| | Classroom management | Appropriate classroom management | 2 | |
| | | Class discipline was not good* | 2 | |
| | | Punished students during the lesson* | 1 | |
| | | Students disliked being reprimanded* | 2 | |
| | | Unfair treatment* | 2 | |
| | | Overtime* | 5 | 14 |
| | Teaching style | Humorous | 5 | |
| | | Longwinded* | 3 | |
| | | Solemn* | 3 | 11 |
| Suggestion | | Strengthen teaching skills and foster attitude | 21 | |
| | | Complete lessons on time | 1 | |
| | | Enhance interaction | 5 | |
| | | Increase the number of activities | 5 | |
| | | Other suggestions | 6 | 38 |

**TABLE 3 (continued)**

| Area | Subcategory | Item | N | N in Subcategory |
|---|---|---|---|---|
| Others | | Instructors' voice was not loud enough* | 1 | |
| | | Other comments on the instructor | 5 | 6 |
| | | **Total (N)** | **361** | |

*Note*: Twenty raw descriptors were randomly selected and coded for examining intra- and inter-rater reliability. The colleagues had to code the randomly selected descriptors into four categories (i.e., positive, neutral, negative, unable to judge) without knowing the original codes given.

* Negative comments.

teaching process, with both positive comments (e.g., facilitate students' learning) and negative comments (e.g., unclear explanation). The percentages of intrarater agreement were 100 and 100%, and those of inter-rater agreement percentage were 100 and 80%.

As shown in Table 4, there were a total of 269 items indicating the areas of improvement that should be made in the program, which were classified into five categories (i.e., program arrangement, program content, process of program implementation, comment on instructors, and others). Most of the respondents suggested that the "program content or format needs to be strengthened" (N = 119, e.g., add more games and activities) and pointed out that the "time issues" (N = 56) in program arrangement should be dealt with (e.g., prolong the duration of each session, program content was too packed). The percentages of intrarater agreement were 90 and 100%, and those of inter-rater agreement percentage were 85 and 95%.

With reference to the total number of responses extracted from the evaluation reports (N = 1,503), it was observed that most of the responses in Table 1–3 could be regarded as positive responses, reflecting the positive evaluation of the program participants. In short, the findings gave support to the conclusion that perceptions of the participants were positive in nature.

## DISCUSSION

The qualitative findings based on the open-ended questions in the present study are generally positive in nature. Including the 40 negative responses in Table 3, and assuming that the "suggestions for refinement" responses in Table 3 and Table 4 are negative responses, they constituted only 23.1% of the total responses. In conjunction with the quantitative subjective outcome evaluation findings reported by Shek and Ma[19], the available evidence suggests that the perceptions of the participants of the program, instructor, and effectiveness of the program were positive. This conclusion is justified based on the principle of triangulation of data types, where both quantitative and qualitative findings point to the positive nature of the perceptions of the program participants.

Besides triangulating the quantitative subjective outcome evaluation findings, the qualitative findings based on the present study are also important as far as program improvement is concerned. Based on the findings outlined in Table 4, the views of the respondents on how the program could be improved are gathered, which provide important pointers for the possible improvement of the program. According to the utilization-focused evaluation paradigm[23], it is important to take the views of the stakeholders into account. The present findings obviously serve this purpose. For the "time issues" raised by the students, it should be noted that it would depend much on the skills of the workers in delivering the teaching units. In fact, it is a basic tension in schools in Hong Kong on how much time in the formal curriculum should be "sacrificed" for positive youth development programs. With reference to the "content issues" raised by the students, it is argued that while the use of varied learning activities would help students to learn, the extent to which games should be used is a matter of balance and judgment based on empirical research findings.

**TABLE 4**
**Summary of the Students' Comments on the Question "Which Aspects of the Program should be Improved?"**

| Area | Subcategory | Item | N | N in Subcategory |
|---|---|---|---|---|
| Program arrangement | Time issues | Duration of program was too long | 2 | |
| | | Shorten the duration of each session | 11 | |
| | | Prolong the duration of each session | 18 | |
| | | Poor time arrangement | 1 | |
| | | Program should be carried out in the normal school timetable | 7 | |
| | | Complete lessons on time | 4 | |
| | | Program content was too packed | 13 | 56 |
| | Number of sessions | Too many sessions | 1 | |
| | | Increase the number of sessions | 5 | 6 |
| | Arrangement of program implementation | Boys and girls should be taught separately | 1 | |
| | | Change the grouping methods | 1 | |
| | | Let more students participate in the activities | 1 | |
| | | Improve classroom environment | 4 | 7 |
| Program content | Program content/format needs to be strengthened | Add more games | 27 | |
| | | Add more activities | 29 | |
| | | Increase reward | 1 | |
| | | Add more outdoor activities | 13 | |
| | | Arrange more time for group discussion | 4 | |
| | | Add more topics | 4 | |
| | | Use more visual teaching materials | 8 | |
| | | Arrange more "free time" | 1 | |
| | | Curriculum content should be more in depth, and close to daily life and trend | 6 | |
| | | Activities should be more diversified | 8 | |
| | | Curriculum and games could be more attractive | 18 | 119 |
| | Program content/format needs to be weakened | Reduce activities that required handwriting | 1 | |
| | | Trim the activities | 1 | |
| | | Decrease the quantity of questionnaires | 2 | |
| | | Decrease the quantity of worksheets | 11 | |
| | | Reduce group discussion | 1 | |
| | | Trim the topics | 2 | 18 |
| | Other comment | Dissatisfied with "Growth Puzzles" | 7 | 7 |
| Process of program implementation | Comment on the process | Should have more fun | 18 | |
| | | Interaction should be increased | 7 | |
| | | Let students play the role of instructor | 1 | 26 |
| | Discipline | Students should be quiet during lessons | 3 | |
| | | Improve class discipline | 3 | 6 |
| Comment on instructors | Teaching skills | Instructors should be more dynamic | 5 | |
| | | Instructors' teaching skills should be improved | 2 | 7 |

**TABLE 4 (continued)**

| Area | Subcategory | Item | N | N in Subcategory |
|---|---|---|---|---|
| Others | | No need to improve | 4 | |
| | | No specific suggestion | 5 | |
| | | Other comments | 8 | 17 |
| | | **Total (N)** | **269** | |

*Note*: Twenty raw descriptors were randomly selected and coded for examining intra- and inter-rater reliability. The colleagues had to code the randomly selected descriptors into four categories (i.e., program arrangement, program content, process of program implementation, comment on instructors) without knowing the original codes given.

The present study clearly demonstrates the possibility of using subjective outcome evaluation findings collected by school and social work units. Nevertheless, it is noteworthy that there are several alternative explanations of the present findings. First, because the data were reconstructed from the reports submitted by the schools, the unit of analysis was the schools rather than the individual program participants. In other words, only the consolidated qualitative findings rather than the raw qualitative findings were analyzed. Second, as the qualitative data collected were written comments, there was no way to have further exchanges with the program participants to gain a more in-depth understanding of the subjective worlds of the program participants. In other words, the qualitative data collected could only be regarded as crude data only.

Third, while the present findings are interpreted in terms of the positive program effects and experiences of the program participants, several alternative explanations are present. The first alternative interpretation is that the students responded positively because they were afraid of punishment if they did not say good things about the program. However, this alternative explanation can be partially dismissed because the students responded in an anonymous manner. The second alternative interpretation is that there was “demand characteristic” of the respondents. However, this alternative explanation could be partially dismissed because negative comments were observed (particularly those reported in Table 4) and the students responded in an anonymous manner.

The third alternative is the “beauty of the beholder side” argument which suggests that the positive responses are the results of the positive biases and expectations of the researchers. However, the intra- and inter-rater reliability with independent raters suggests that this possibility is not high. In addition, with reference to the 12 principles that should be maintained in a qualitative evaluation study[20], the present study fulfilled 10 principles, including explicit statement of the philosophical base of the study (Principle 1), justifications for the number and nature of the participants of the study (Principle 2), detailed description of the data collection procedures (Principle 3), discussion of the biases and preoccupations of the researchers (Principle 4), description of the steps taken to guard against biases or arguments that biases should and/or could not be eliminated (Principle 5), inclusion of measures of reliability, such as inter- and intrarater reliability (Principle 6), inclusion of measures of triangulation in terms of researchers and data types (Principle 7), consciousness of the importance and development of audit trails (Principle 9), consideration of alternative explanations for the observed findings (Principle 10), and clear statement of the limitations of the study (Principle 12). In conclusion, the present study based on qualitative data of subjective outcome evaluation provides additional support for the effectiveness of the Tier 1 Program of the Project P.A.T.H.S. in Hong Kong.

## ACKNOWLEDGMENTS

The preparation for this paper and the Project P.A.T.H.S. were financially supported by the Hong Kong Jockey Club Charities Trust.
